# Value of Intraoperative Pancreatoscopy in Patients Undergoing Resection for Main-Duct Intraductal Papillary Mucinous Neoplasm: Thirty-Day Follow-Up of a Prospective International Study

**DOI:** 10.1097/AS9.0000000000000638

**Published:** 2026-01-02

**Authors:** Urban Arnelo, Roberto Valente, Marc G. Besselink, Christian Max Schmidt, Stuart Sherman, Guduru Venkat Rao, Richard A. Burkhart, Yi Miao, Sohei Satoi, Rogier P. Voermans, Shreeyash Modak, Steven Edmundowicz, Olivier R. Busch, Kelly J. Lafaro, Jishu Wei, Joyce A. Peetermans, Matthew J. Rousseau, Marco Del Chiaro, for the Intraoperative Pancreatoscopy Study Group, Urban Arnelo

**Affiliations:** Umeå University, Umeå, Sweden; Umeå University, Umeå, Sweden; Umeå University, Umeå, Sweden; Umeå University, Umeå, Sweden; Umeå University, Umeå, Sweden; Amsterdam UMC, Amsterdam, the Netherlands; Amsterdam UMC, Amsterdam, the Netherlands; Amsterdam UMC, Amsterdam, the Netherlands; Amsterdam UMC, Amsterdam, the Netherlands; Amsterdam UMC, Amsterdam, the Netherlands; Amsterdam UMC, Amsterdam, the Netherlands; Indiana University School of Medicine, Indianapolis, IN; Indiana University School of Medicine, Indianapolis, IN; Asian Institute of Gastroenterology, Hyderabad, India; Asian Institute of Gastroenterology, Hyderabad, India; Asian Institute of Gastroenterology, Hyderabad, India; Johns Hopkins Hospital, Baltimore, MD; Johns Hopkins Hospital, Baltimore, MD; Johns Hopkins Hospital, Baltimore, MD; Johns Hopkins Hospital, Baltimore, MD; Johns Hopkins Hospital, Baltimore, MD; The First Affiliated Hospital with Nanjing Medical University, Nanjing, China; The First Affiliated Hospital with Nanjing Medical University, Nanjing, China; Kansai Medical University, Hirakata City, Osaka, Japan; Kansai Medical University, Hirakata City, Osaka, Japan; Kansai Medical University, Hirakata City, Osaka, Japan; University of Colorado, Denver, CO; Boston Scientific Corporation, Marlborough, MA; Boston Scientific Corporation, Marlborough, MA; From the * Department of Diagnostics and Intervention, Surgery, Umeå University, Umeå, Sweden; † Department of Surgery, Amsterdam UMC, University of Amsterdam, Amsterdam, the Netherlands; ‡ Cancer Center Amsterdam, VU University, Amsterdam, the Netherlands; § Department of Surgery, Indiana University School of Medicine, Indianapolis, IN; ∥ Division of Gastroenterology and Hepatology, Indiana University School of Medicine, Indianapolis, IN; ¶ Asian Institute of Gastroenterology, Hyderabad, India; # Division of Hepatobiliary and Pancreatic Surgery, Johns Hopkins Hospital, Baltimore, MD; ** Department of General Surgery, Pancreas Center, The BenQ Medical Center Affiliated of Nanjing Medical University, Nanjing, China; †† Department of Surgery, Kansai Medical University, Hirakata City, Osaka, Japan; ‡‡ Department of Gastroenterology and Hepatology, University of Amsterdam, Amsterdam, the Netherlands; §§ Division of Surgical Oncology, Department of Surgery, University of Colorado, Anschutz Medical Campus, Denver, CO; ∥∥ Boston Scientific Corporation, Marlborough, MA; ¶¶ Department of General Surgery, Pancreas Center, The First Affiliated Hospital of Nanjing Medical University, Nanjing, China.

**Keywords:** IPMN, high-grade dysplasia, pancreatoscopy, pancreatic surgery, pancreatic cancer

## Abstract

**Objective:**

To report 30-day results from an ongoing 5-year study of intraoperative pancreatoscopy (IOP) in patients undergoing pancreatic surgery for main pancreatic duct (MPD) intraductal papillary mucinous neoplasm (IPMN).

**Background:**

IOP may detect skip lesions and objectify disease extent during surgery for IPMN with MPD involvement, but prospective multicenter data are lacking.

**Methods:**

Prospective international study including patients with IPMN and MPD diameter >5 mm on preoperative imaging, scheduled for surgery at 8 centers in 6 countries. Primary endpoint was the rate of detection by IOP of discontinuous (skip) lesions along the MPD. Secondary endpoints included IOP technical success, influence of IOP findings on surgical plan, and related serious adverse events (SAEs).

**Results:**

Among 100 patients undergoing IOP, skip lesions were documented in 13 (13.0%). IOP was technically successful in 91 (91.0%). In 41 patients (41.0%), IOP findings were reported by the surgeon to be impactful by confirming the absence of lesions in a dilated MPD and hence sparing parenchyma in 26 (26%), extending the initial resection in 12 (12%), or changing the type of partial pancreatectomy in 3 (3%). There were no IOP-related SAEs. Surgery-related SAEs were reported in 27 (27%) patients.

**Conclusions:**

IOP identified skip lesions in 13% of patients undergoing surgery for IPMN with MPD involvement. IOP influenced the surgical plan in 41% of patients. There were no IOP-related SAEs. Longer follow-up is ongoing. ClinicalTrials.gov number NCT03729453.

## Graphical abstract

**Figure:**
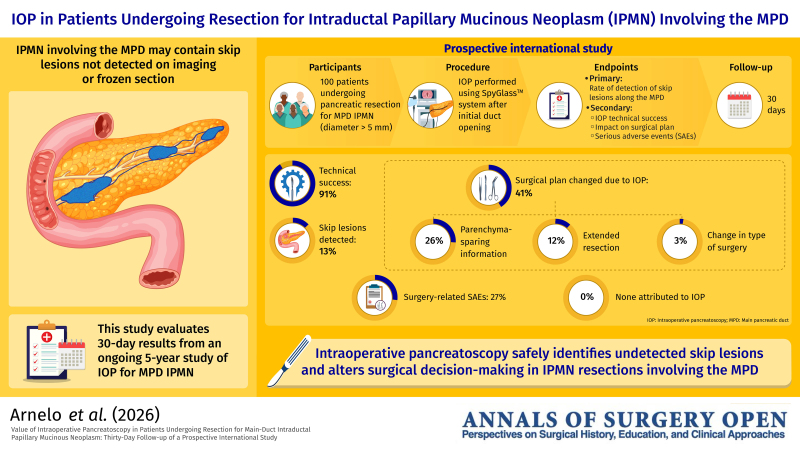

## INTRODUCTION

Intraductal papillary mucinous neoplasm (IPMN) with involvement of the main pancreatic duct (MPD) is often managed with surgical resection due to the higher risk of harboring malignancy [high-grade dysplasia (HGD) and cancer] when compared with branch duct IPMN.^1,2^ One of the major challenges during surgery for main duct-IPMN and mixed type-IPMN is the risk of having potential discontinuous (“skip”) lesions along the MPD, distant from the surgical resection margin. The exact prevalence of IPMN skip lesions in the MPD is unknown, but previous surgical series have suggested their presence in up to 21% of patients,^3^ potentially at least in part, explaining the risk of postsurgical IPMN recurrence. A second major challenge during surgery for MPD-IPMN is to correctly interpret whether or not the presence of a dilated “upstream” MPD may be due to remnant IPMN or due to secondary dilation of the duct related to “downstream” mucus produced by the IPMN.^4^

Intraoperative pancreatoscopy (IOP) has been suggested as a diagnostic tool to improve characterization of IPMN during surgery, ultimately potentially leading to improved intraoperative decision-making.^5–7^ A 2023 single-center retrospective cohort study of 46 patients reported that the combination of operator assessment during IOP and frozen section provided the highest score in sensitivity and specificity, 85.7%, 95% confidence interval (42.1–99.6) and 92.3%, 95% confidence interval (63.9–99.8), respectively with overall best diagnostic precision for detection of pathological tissue in the remnant pancreas.^5^

IOP may provide detailed imaging of the MPD, potentially identifying skip lesions that would otherwise remain undiagnosed in the setting of intraoperative frozen sections negative for IPMN or to confirm the absence of IPMN in a dilated MPD. To better characterize the clinical merit and safety of IOP, we performed a prospective study in patients with suspected IPMN with MPD involvement who were scheduled for surgery shortly after enrollment. Here we report 30-day results of IOP in patients undergoing pancreatic surgery for MPD IPMN. Five-year follow-up of study patients is ongoing.

## METHODS

### Study Design

We conducted a prospective, multicenter, international cohort study to evaluate the added value of IOP in patients undergoing pancreatic resection for the treatment of IPMN with MPD involvement, including the impact of the detection of discontinuous (skip) lesions in the pancreatic remnant when a partial pancreatectomy was planned. The devices used in the study were the SpyScope DS and DS II Access and Delivery Catheter, SpyGlass Discover Digital Catheter, SpyScope DS Digital Controller, and the SpyBite and SpyBite Max Biopsy Forceps (Boston Scientific Corporation, Marlborough, Massachusetts). All centers obtained approval from their respective local ethics committees or institutional review boards.

### Patient Population

Eligible patients had an MPD diameter of >5 mm on preoperative magnetic resonance imaging or computed tomography and were scheduled for surgery for suspected Main Duct IPMN or Mixed Type IPMN within 6 weeks of enrollment. Exclusion criteria included age less than 18 years and pregnancy. All patients provided written informed consent to participate in the study.

### Endoscopic Procedure

Pancreas resections were performed by experienced surgeons through an open surgical or a laparoscopic approach based on the investigator’s recommendation and standard of practice. Laparoscopic cases could be performed robotically, also at the discretion of the surgeon. The intraoperative endoscopic procedure was performed as previously described by Arnelo et al^5^ by surgeons with or without the help of an expert interventional endoscopist. After initial division of the pancreatic parenchyma at the neck of the gland, the pancreatic duct was opened and allowed for easy cannulation (insertion) of the pancreatoscope. Visualization with IOP was performed at least once on the remnant pancreatic duct after the initial transection. Operators recorded the length of the inspected MPD. Impressions of the MPD in the remnant portion of the pancreas were recorded, including normal duct, discontinuous (skip) lesions, macroscopic findings on visual inspection, and other relevant details such as papillary lesions, vessels, hemorrhagic/necrotic tissue, mucinous discharge, stricture, and/or stones. Based on the investigator’s experience, the IOP findings were classified as normal, suspicious for malignancy, or malignant.

The resected lesion margin could be extended multiple times at the surgeon’s discretion, depending on the frozen section analysis, the macroscopic impression of IOP, anatomy, or the life expectancy/comorbidities of the patient. The operator reported whether visualization with IOP of the remnant duct was deemed useful for patient management. No interventions were recommended by the study protocol. The decision to use or not to use and how to use the information acquired during the examination was fully left to the surgeon.

Frozen section details on the resection margin were recorded during the procedure when frozen section histology was performed. In addition, if a discontinuous (skip) lesion or any other suspicious findings were made in the remnant duct, the surgeon could obtain pancreatoscopy-guided targeted tissue biopsies if desired and/or could decide to extend the resection margin to achieve more radical surgery.

### Study Visits

Postoperative in-hospital follow-up data were recorded at least once before hospital discharge. The subsequent patient follow-up contact was conducted at 4 weeks ± 2 weeks after the index surgery. At each time point, adverse events as ongoing and/or as extracted from medical records were recorded and evaluated based on clinical presentation, postoperative imaging, and blood work per local standard of practice. IPMN lesion recurrence, new metastatic disease, or progression of disease on postoperative imaging were recorded. Subjects were considered lost-to-follow-up if there were 3 unsuccessful attempts to contact them.

### Primary Endpoint

The primary endpoint was the rate of detection of discontinuous (skip) lesion(s) along the MPD of patients with IPMN during pancreatic resection using IOP based on visual impression of IPMN and/or pancreatoscopy-guided biopsies.

### Secondary Endpoints

The secondary endpoints of relevance to this report were (1) technical success of IOP, defined as ability to advance the pancreatoscope along the entire length of dilated MPD, visualize skip lesion(s) where applicable, and obtain an intraductal biopsy when desired, (2) final diagnosis of IPMN documented in an open-text field in the pathology section of the data collection form, (3) influence of IOP on the surgical resection plan, and (4) serious adverse events (SAEs) related to the IOP procedure and/or pancreatoscope reported according to the Clavien-Dindo Classification.^8^ Surgeons were required to specify whether the SAE was “not related” or had an “unlikely,” “possible,” “probable,” or “causal” relationship to pancreatoscopy and/or surgical procedure. The relatedness of any adverse event as reported by each investigational site was used for the analysis of this safety endpoint.

### Statistical Analysis

Baseline characteristics, medical history, outcome measures, and adverse events were summarized using mean, median, standard deviation, and range for continuous variables (eg, age and procedure times), and proportions for categorical variables (eg, sex, race, and organ failure score).

## RESULTS

### Patient Characteristics

Overall, 100 patients met the eligibility criteria and were enrolled. The cohort had a mean age of 69 ± 9 (range 41–88) years, with 58 (58.0%) male participants (Table 1). Ten patients had a preoperative endoscopic retrograde cholangiopancreatography, including 9 who received a pancreatic stent and 8 whose stents were still in place at the time of surgery. The indication for surgery was MPD dilation of greater than 10 mm on imaging in 58 patients (58.0%), while 42 (42.0%) patients had MPD dilation of 5–9.9 mm. Suspected or confirmed cancer was also listed as an indication for surgery in 6 (6.0%) patients. The 2018 European guidelines^9^ were used to determine the need for surgery in 59 (59.0%) patients, while the 2017 International guidelines^10^ were used for the remaining 41 (41.0%) patients. Ninety-eight (98.0%) patients completed the 30-day follow-up visit. One of the 2 (2.0%) who missed the 30-day visit died on day 13 of postsurgical septic shock (see “Procedure-Related Serious Adverse Events at 30 days”).

**TABLE 1.** Patient Baseline Characteristics, Preoperative Imaging, and Presurgical Planning (N = 100)

Baseline Characteristic	% (n/N), Mean ± SD (n) or Median (n) (Range)
Age	68.9 ± 8.8
Male	58.0 (58/100)
Relevant medical conditions/comorbidities	
Diabetes	31.0 (31/100)
Acute pancreatitis	10.0 (10/100)
Chronic pancreatitis	7.0 (7/100)
Prior stenting of MPD	9.0 (9/100)
Stent in place at time of surgery	8.0 (8/100)
Stent not in place at time of surgery	1.0 (1/100)
Subject using aspirin or NSAIDs	16.0 (16/100)
Subject using anticoagulants	24.0 (24/100)
Indication for surgery	
Main pancreatic duct dilation of >5–9 mm, on imaging	42.0 (42/100)
Main pancreatic duct dilation of ≥10 mm, on imaging	58.0 (58/100)
Preoperative imaging	
Type of imaging performed	
CT	65.0 (65/100)
EUS ± FNA (fine needle aspiration)	21.0 (21/100)
MRI	9.0 (9/100)
MRCP	3.0 (3/100)
ERCP	1.0 (1/100)
Pancreatoscopy	1.0 (1/100)
Ductal involvement	
Main duct	99.0 (99/100)
Branch duct	38.0 (38/100)
Mean radiological length of dilated duct (mm)	80.4 ± 53.5(64)
Mean radiological diameter of dilated duct (mm)	16.2 ± 18.2 (94)
Diagnosis based on pre-op imaging and/or clinical presentation	
Main duct-IPMN	38.0 (38/100)
Mixed IPMN	56.0 (56/100)
Cancer (unspecified)	5.0 (5/100)
Malignant IPMN	1.0 (1/100)
Surgical planning	
Surgical procedure planned	
Pancreatoduodenectomy	82.0 (82/100)
Standard pancreatoduodenectomy (PD)	74 (74/100)
Pylorus-preserving pancreatoduodenectomy (PPPD)	8.0 (8/100)
Left pancreatectomy	14.0 (14/100)
Total pancreatectomy	4.0 (4/100)
Preoperative American Society of Anesthesiologists status	
ASA I	17.0 (17/100)
ASA II	36.0 (36/100)
ASA III	40.0 (40/100)
Not documented	7.0 (7/100)
Preoperative laboratory values, median	
Amylase, U/L	44.0 (48) (0.1,982.0)
CA 19-9, U/mL	24.2 (78) (1.0,2173.0)
Hemoglobin g/dL	11.7 (92) (6.8,16.2)
Platelet count (platelets/μL)	255,000 (85) (120,000, 635,000)
INR	1.0 (80) (0.9,12.2)

CT indicates computed tomography; ERCP, endoscopic retrograde cholangiopancreatography; EUS, endoscopic ultrasound; MRI, magnetic resonance imaging; MRCP, magnetic resonance cholangiopancreatography; NSAID, nonsteroidal anti-inflammatory drug; SD, standard deviation.

### Surgical Procedures

Surgical procedures were open in 71 (71.0%), robotic in 23 (23.0%), and laparoscopic in 6 (6.0%) patients. Final performed surgeries were pancreatoduodenectomy in 74 (74%), left pancreatectomy in 15 (15.0%), and total pancreatectomy in 11 (11.0%) patients (Table 2).

**TABLE 2.** Surgical Procedure Details (N = 100)

	% (n/N)
Initial planned surgical course modified based on IOP findings	41.0 (41/100)
Parenchyma-sparing	26.0 (26/100)
Extension of resection	12.0 (12/100)
Change in type of surgery	3.0 (3/100)
Final surgical procedure	
Open	71.0 (71/100)
Laparoscopic	6.0 (6/100)
Robotic	23.0 (23/100)
Surgical procedure performed	
Pancreatoduodenectomy	75.0 (75/100)
Standard pancreatoduodenectomy (PD)	56.0 (56/100)
Pylorus-preserving pancreatoduodenectomy (PPPD)	19.0 (19/100)
Left pancreatectomy	14.0 (14/100)
Total pancreatectomy	11.0 (11/100)
No additional procedures performed	62.0 (62/100)

### Primary Endpoint: Skip Lesions Along the MPD

**FIGURE 1.**
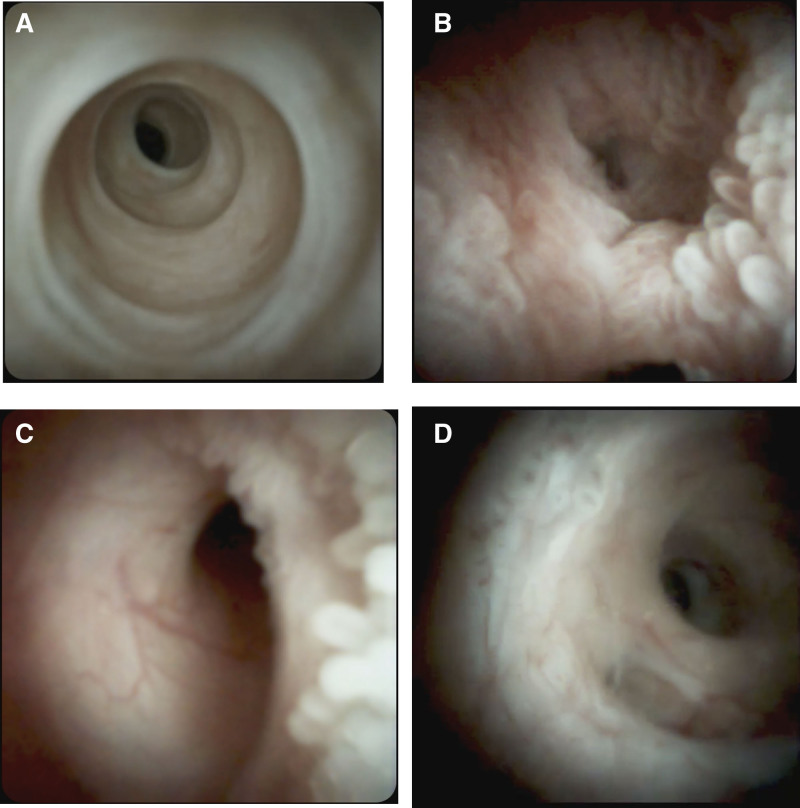
Intraoperative pancreatoscopy in patients undergoing pancreatic resection for the treatment of intraductal papillary mucinous neoplasm (IPMN) main-duct involvement. A, Example of a healthy main pancreatic duct (main pancreatic duct). The epithelial lining is smooth and symmetrical without pathology. B, Extensive circumferential IPMN lesion in the pancreatic head. C and D, Examples of skip lesions (1) An island with fingerlike papillae (12 to 5 o’clock), (2) A small island of subtle egglike papillae (11 o’clock).

Discontinuous skip lesions were identified in 13 (13.0%) patients during IOP (Fig. 1). In 9 of 13 cases (69%), the pre-IOP planned surgery was pancreatoduodenectomy, which was changed to total pancreatectomy in 4 of 9 patients, remained unchanged in 3 of 9, and underwent an extended resection line in 2 of 9 patients. In 3 of the 13 patients (23%) in whom a skip lesion was identified by IOP, the pre-IOP surgical plan was for a total pancreatectomy, which was indeed performed. In one of the 13 patients (8%), the pre-IOP surgical plan was a left pancreatectomy, which remained unchanged.

Eight patients with skip lesions had MPD diameter ≥10 mm and 5 had MPD diameter between 5 mm and 9 mm at baseline imaging. In 10 of 13 patients (77%) an intraoperative frozen section was analyzed.

### Secondary Endpoints

#### Technical Success

IOP was technically successful in 92 (92.0%) patients. The mean depth of pancreatoscope insertion into the MPD was 78 ± 34 mm. Eight patients (8.0%) did not meet the criteria for technical success because the scope could not be advanced as far as intended, but the endoscopists reported that the macroscopic appearance of the tissue could still be assessed.

#### Final Diagnosis of IPMN

A final diagnosis of IPMN based on histopathology of the specimen was documented for 61 (61%) patients (Table 3). An additional 24 (24%) had a diagnosis of adenocarcinoma that did not rule out concomitant presence of IPMN. Fourteen (14%) of patients had non-IPMN, non-adenocarcinoma final diagnoses, and 1 (1%) did not have final histopathology.

**TABLE 3.** Final Diagnosis Based on Histopathology of Specimen

Histopathology Result	% (n/N)
IPMN	61 (61/100)
With high-grade dysplasia	60 (16/100)
With intermediate-grade dysplasia	1 (1/100)
With low-grade dysplasia	18 (18/100)
IPMN, not otherwise specified (NOS)	26 (26/100)
PDAC/carcinoma	24 (24/100)
Pancreatic adenocarcinoma	16 (16/100)
Adenocarcinoma, NOS	4 (4/100)
Invasive mucinous carcinoma, not IPMN	2 (2/100)
Ampullary adenocarcinoma	1 (1/100)
Duodenal papillary adenocarcinoma	1 (1/100)
Non-IPMN, non-carcinoma	14 (14/100)
Low-grade dysplasia	3 (3/100)
Serous cystadenoma	3 (3/100)
PanIN	2 (2/100)
High-grade dysplasia	1 (1/100)
Invasive neuroendocrine tumor grade I	1 (1/100)
Chronic pancreatitis	1 (1/100)
No tumor present, negative histopathology	3 (3/100)
No final pathology	1 (1/100)

#### Influence of IOP on Surgical Resection Plan

Before surgery, the surgical plan was pancreatoduodenectomy in 82 (82%), left pancreatectomy in 14 (14%), and total pancreatectomy in 4 (4%) patients. Pancreatic surgeons reported that IOP had impacted surgical decision-making in 41 (41%) and had no impact in 59 (59%) patients.

**Figure 2.**
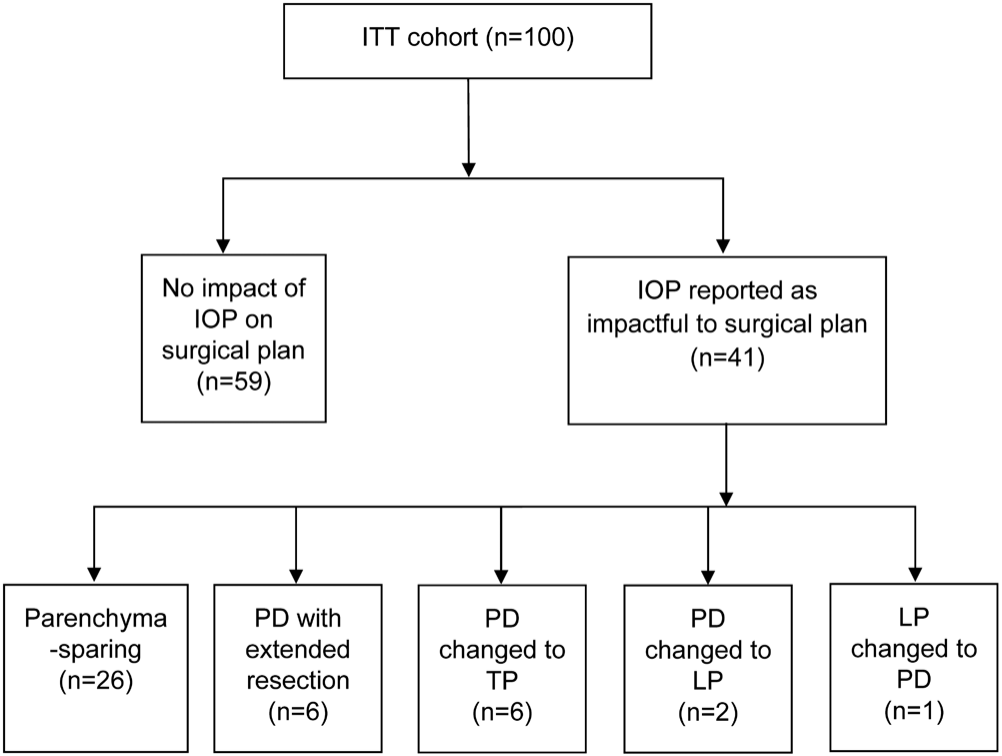
IOP impact on surgical plan. ITT indicates intention to treat; PD, pancreatoduodenectomy; TP, total pancreatectomy; LP, left pancreatectomy.

In the 41 patients in whom IOP was reported impactful, the impact was reported as confirming that there were no concerning findings in the dilated duct in the remnant of the partial pancreatectomy and hence IOP having provided parenchyma-sparing information in 26 (26%) patients. In 12 (12%) patients, IOP was reported to have led to extension of resection, notably in 6, the initial division was altered to extend the resection within a pancreatoduodenectomy and in 6 patients the pancreatoduodenectomy was converted to total pancreatectomy. In 3 (3%) patients, IOP was reported to have impacted the decision to change from pancreatoduodenectomy to left pancreatectomy (2) or from left pancreatectomy to pancreatoduodenectomy (1). Figure 2 shows an overview of the IOP impact on surgical plan.

Abnormal IOP findings were reported as small papillary lesions without vessels, larger papillary lesions with vessels, hemorrhagic or necrotic tissue, tortuous vessels, mucinous discharge, stricture not seen on preoperative noninvasive imaging, and stones. In patients in whom IOP was reported as impactful, the mean number of such abnormal IOP findings was 1.5 ± 1.5, and the number of patients with at least 1 abnormal IOP finding was 68.3% (28/41). In patients in whom IOP was reported as not impactful, the mean number of IOP findings was 1.1 ± 1.4, with 49.2% (28/59) patients having at least 1 abnormal IOP finding.

#### Impact of IOP Findings and Frozen Section Histology

Intraoperative frozen sections were not collected in several study cases, mostly from 1 participating center. In addition, in 2 cases, a frozen section was collected but discarded before being analyzed because the patient was to undergo a total pancreatectomy. In total, IOP and analysis of an intraoperative frozen section were available for 65 of 100 patients (65%).

In this subset of 65 patients, 57 had no skip lesion observation on IOP and 8 had skip IPMN lesions on IOP. In 2 of the 8 patients (25%), the resection was extended—1 pancreatoduodenectomy with an extended resection line and 1 pancreatoduodenectomy converted to total pancreatectomy—in the setting of the margin frozen section being negative for cancer or HGD.

### Procedure-Related SAEs at 30 Days

Twenty-seven (27.0% of 100) patients reported 47 procedure-related SAEs (Table 4). The SAEs were (one or more of the following in each patient): abdominal pain or delayed gastrointestinal transit (15 patients), leakages [12 patients who had pancreatic leak/fistula (9), biliary leak (2), hepaticojejunostomy and chyle leak (both in 1 patient)], infection or inflammation (7 including 1 patient with bacteremia and fatal septic shock), fluid collection (gastrointestinal in 5 patients and pleural effusion in 1), or blood-related (2 patients). No SAEs were reported as related to the IOP device or procedure. All 47 reported SAEs were related to surgery with Clavien-Dindo classification ≥ III in 16 patients, including 1 Clavien-Dindo V.

**TABLE 4.** Surgical Procedure-Related Serious Adverse Events (SAEs) Among All Patients (N = 100)

	No. SAEs	No. Patients
Any SAE	47	27.0% (27/100)
Abdominal pain or delayed gastrointestinal transit	17	15.0% (15/100)
Leaks	14	12.0% (12/100)
Infection or inflammation	7	7.0% (7/100)
Fluid collection	6	6.0% (6/100)
Blood- or electrolyte-related disorder	3	2.0% (2/100)

## DISCUSSION

In this first prospective multicenter study on IOP in patients undergoing surgery for IPMN and an MPD diameter of >5 mm on preoperative imaging, IOP found skip lesions in 13% and led to modification of the original planned surgical course in 41% of patients, most often regarding the extent of parenchymal resection, including 12% of patients in whom the initially planned resection was extended. There were no IOP-related SAEs.

A 2021 meta-analysis emphasized that MPD dilation >5 mm should trigger consideration of surgical resection^11^ due to the risk of HGD and malignancy. The largest series of resected IPMN^12^ reported that the best threshold for risk of HGD or cancer seemed to be MPD dilation of ≥7 mm. Accordingly, current guidelines^9,10^ recommend surgical resection for IPMNs with mural nodules (especially if diameter >5 mm) or with involvement of the MPD (especially with MPD diameter >10 mm), while surgical resection is considered in only a minority of patients with branch duct IPMN because of significantly lower cancer risk in these patients.^13^ The most important determinant of outcome in the management of patients with IPMN is the presence of an associated invasive carcinoma, since patients with completely resected noninvasive IPMNs have a 5-year survival of >90%, whereas half of patients with an associated invasive carcinoma die from their disease.^14^ Therefore, the focus of diagnostic efforts is to identify HGD, before invasive carcinoma develops. Accurate characterization of IPMN lesions is important to identify surgical candidates, while surgery can improve outcomes, and to determine an appropriate follow-up schedule for the majority of patients for whom IPMN findings are benign.^15^ Due to the indolent nature of most IPMNs, progression may occur after 5 years or more of follow-up, and some patients undergo surveillance for over 10 years.^16^ Skip lesions raise concerns for the possibility of recurrence after partial pancreatectomy with a normal transection margin.^17^ In our study, skip lesions were found in 8/58 (14%) patients with MPD >10 mm and 5/42 (12%) patients with MPD 5–9 mm. To detect skip lesions, the only reported techniques are cytology of pancreatic juice harvested in the remnant in addition to frozen section, and IOP with staged biopsies.^5,6,17–19^ However, cytology of pancreatic juice is not able to localize precisely the possible location of the lesion.

IOP was first reported in 1998 as a safe and effective diagnostic intervention that showed accuracy superior to endoscopic retrograde pancreatography and endoscopic ultrasound in the detection of intraductal mural nodules in a small sample of mucin-producing pancreatic tumors.^20^ A 2022 meta-analysis of the role of pancreatoscopy in the diagnostic work-up of IPMN noted that data on IOP are scarce, but small studies suggest its use can alter surgical management.^5,21^ Recently, the use of IOP has also been described during robot-assisted pancreatic surgery (ie, both pancreatoduodenectomy and left pancreatectomy).^18^

The use of IOP in conjunction with frozen section pathology is of special interest to determine appropriate surgical margins.^5,22^ Frozen section is an important intraoperative diagnostic tool, especially for margin assessment in the pancreatic neck margin, where accuracy and utility have been shown to be very high.^23^ While positive margins definitively portend worse survival in nearly all studies of pancreatoduodenectomy,^24^ the impact of their resection remains debated because some studies have^24,25^ but others have not^26^ reported a survival benefit with resection of positive margins. For this reason, not all centers routinely perform frozen section pathology, also in the current study. The 2018 European evidence-based guidelines on pancreatic cystic neoplasms recommended that pancreatoscopy may be used in selected patients to investigate the location and extent of main-duct IPMN, to differentiate chronic pancreatitis from main-duct IPMN, and combined with frozen section to establish the extent of IPMN involvement of the MPD and assist surgical decision-making about the extent of resection required.^9^

Compared to a 2023 single-center prospective study in 46 patients with a mean of 34.7 months of follow-up,^5^ the current study showed similar success in exploring the entire dilated duct (93.4% in the pilot study vs 91% in the current study) and similar mean length of inspected duct (7.5 cm vs 7.8 cm, respectively). The current study had a lower proportion of patients with skip lesions (13% in the current study vs 43.4% in the pilot study) and a lower proportion for whom IOP was felt to change the operative course (41% vs 65%, respectively). Both of these were observational real-life studies aimed at characterizing IOP visual findings, confirming safety, and estimating the “added value” of IOP findings in patients who had surgery due to suspicion of IPMN. A case-control study with a matched non-IOP comparator would probably add more information, while an randomized controlled trial would probably harbor a less real-life view, and thus less applicability in real-life clinical settings.

Our study results should be interpreted considering several limitations. First, this was an observational study without a comparator. No guidance was offered by the study protocol pertaining to the preoperative work-up or whether to alter the initial surgical plan based on IOP findings; therefore, the results could be influenced by the surgeons’ different practice and experience. Second, consecutive patient recruitment was not possible due to the multicenter setting of the study, in which different operators have had impact in determining the indication to include the patient. For this reason, the study population and their results may not be typical of all IPMN patients undergoing pancreatic resection. Third, the timing of the IOP visualization and frozen sections, eg, before versus after extension of margins where applicable was not standardized, once again underlying potential differences of clinical praxis at different centers. Fourth, SpyBite biopsy’s diagnostic yield was not among the endpoints of the study. Fifth, an intraoperative frozen section was not performed in some patients. This limits the potential interpretation of the clinical merit of combining IOP and frozen section analysis. Sixth, the 5-year follow-up of the study patients is still ongoing. Therefore, IPMN recurrence is not addressed in this primary endpoint and procedural findings report. The main strengths of our study include being the largest and longest-running prospective study of IOP in IPMN to date. Unlike many previous studies, the patients were limited to IOP, not a mixture of preoperative and IOP. In contrast to preoperative pancreatoscopy, where significant rates of procedure-related pancreatitis have been documented,^5,27,28^ no cases of postoperative acute pancreatitis occurred after our study patients underwent IOP procedures.

## CONCLUSIONS

IOP detected skip lesions in 13% of patients and led to changes in decision-making in 41% of patients with IPMN and MPD involvement undergoing pancreatic surgery. IOP was safe within the limitations of the study. Five-year follow-up is ongoing.

## ACKNOWLEDGMENTS

The authors acknowledge Velina Chavarro, MD, for project management and Boston Scientific Corporation employee Margaret Gourlay, MD, MPH, for providing writing assistance.

Conception and design: U.A., R.V., J.A.P., M.J.R., and M.D.C. Acquisition, analysis, or interpretation of data for the work: all authors. Interpretation of data and drafting of the manuscript: U.A., R.V., M.G.B., J.A.P., M.J.R., and M.D.C. Critical revision of the manuscript for important intellectual content: U.A., R.V., M.G.B., Y.M., S. Satoi, R.P.V., J.A.P., M.J.R., and M.D.C. Final review and approval of the article: all authors. Obtained funding: J.A.P. Administrative, technical, or material support and supervision: U.A. and J.A.P. Justification for inclusion of more than 8 authors: Surgeons and GI endoscopists at 8 medical centers in 6 countries participated in this study of traditional pancreatic surgery with intraoperative pancreatoscopy. This led to a large number of authors. Other physicians were acknowledged because they participated but did not meet authorship criteria. One author (S. Sherman) died during the study period but met authorship criteria for his contributions before then.

Members of the Intraoperative Pancreatoscopy Study Group are as follows: Urban Arnelo, Roberto Valente, Asif Halimi, Oskar Franklin, and Chiara Scandavini, Umeå University, Umeå, Sweden. Marc G. Besselink, Charlotte Leseman, Myrte Gorris, Olivier R. Busch, Freek Daams, and Rogier P. Voermans, Amsterdam UMC, Amsterdam, the Netherlands. Christian Max Schmidt and Stuart Sherman, Indiana University School of Medicine, Indianapolis, IN. Guduru Venkat Rao, Shreeyash Modak, and Sana Fatima Memon, Asian Institute of Gastroenterology, Hyderabad, India. Richard A. Burkhart, Kelly J. Lafaro, Christopher R. Shubert, Jin He, and William R. Burns, Johns Hopkins Hospital, Baltimore, MD. Yi Miao and Jishu Wei, The First Affiliated Hospital with Nanjing Medical University, Nanjing, China. Sohei Satoi, Daisuke Hashimoto, and So Yamaki, Kansai Medical University, Hirakata City, Osaka, Japan. Steven Edmundowicz, University of Colorado, Denver, CO. Joyce A. Peetermans and Matthew J. Rousseau, Boston Scientific Corporation, Marlborough, MA.

Stuart Sherman, MD, Deceased.

## References

[1] Ohtsuka T Fernandez-Del Castillo C Furukawa T. International evidence-based Kyoto guidelines for the management of intraductal papillary mucinous neoplasm of the pancreas. Pancreatology. 2023;24:255–270. 38182527 10.1016/j.pan.2023.12.009

[2] Scholten L van Huijgevoort NCM Bruno MJ. Surgical management of intraductal papillary mucinous neoplasm with main duct involvement: an international expert survey and case-vignette study. Surgery. 2018;S0039-6060:30082–30085. 10.1016/j.surg.2018.01.025 29778250

[3] Eguchi H Ishikawa O Ohigashi H. Role of intraoperative cytology combined with histology in detecting continuous and skip type intraductal cancer existence for intraductal papillary mucinous carcinoma of the pancreas. Cancer. 2006;107:2567–2575. 17054109 10.1002/cncr.22301

[4] Wolske KM Ponnatapura J Kolokythas O. Chronic Pancreatitis or Pancreatic Tumor? A Problem-solving Approach. Radiographics. 2019;39:1965–1982. 31584860 10.1148/rg.2019190011

[5] Arnelo U Valente R Scandavini CM. Intraoperative pancreatoscopy can improve the detection of skip lesions during surgery for intraductal papillary mucinous neoplasia: a pilot study. Pancreatology. 2023;23:704–711. 37336668 10.1016/j.pan.2023.06.006

[6] Navez J Hubert C Gigot JF. Impact of intraoperative pancreatoscopy with intraductal biopsies on surgical management of intraductal papillary mucinous neoplasm of the pancreas. J Am Coll Surg. 2015;221:982–987. 26304184 10.1016/j.jamcollsurg.2015.07.451

[7] Pucci MJ Johnson CM Punja VP. Intraoperative pancreatoscopy: a valuable tool for pancreatic surgeons? J Gastrointest Surg. 2014;18:1100–1107. 24664423 10.1007/s11605-014-2501-9

[8] Clavien PA Barkun J de Oliveira ML. The Clavien-Dindo classification of surgical complications: five-year experience. Ann Surg. 2009;250:187–196. 19638912 10.1097/SLA.0b013e3181b13ca2

[9] European Study Group on Cystic Tumours of the Pancreas. European evidence-based guidelines on pancreatic cystic neoplasms. Gut. 2018;67:789–804. 29574408 10.1136/gutjnl-2018-316027 PMC5890653

[10] Tanaka M Fernandez-Del Castillo C Kamisawa T. Revisions of international consensus Fukuoka guidelines for the management of IPMN of the pancreas. Pancreatology. 2017;17:738–753. 28735806 10.1016/j.pan.2017.07.007

[11] Wu YHA Oba A Beaty L. Ductal Dilatation of >/=5 mm in intraductal papillary mucinous neoplasm should trigger the consideration for pancreatectomy: a meta-analysis and systematic review of resected cases. Cancers (Basel). 2021;13:2031. 33922344 10.3390/cancers13092031 PMC8122854

[12] Del Chiaro M Beckman R Ateeb Z. Main duct dilatation is the best predictor of high-grade dysplasia or invasion in intraductal papillary mucinous neoplasms of the pancreas. Ann Surg. 2020;272:1118–1124. 30672797 10.1097/SLA.0000000000003174

[13] Del Chiaro M Ateeb Z Hansson MR. Survival analysis and risk for progression of intraductal papillary mucinous neoplasia of the pancreas (IPMN) under surveillance: a single-institution experience. Ann Surg Oncol. 2017;24:1120–1126. 27822633 10.1245/s10434-016-5661-x PMC5339331

[14] Adsay V Mino-Kenudson M Furukawa T; Members of Verona Consensus Meeting, 2013. Pathologic evaluation and reporting of intraductal papillary mucinous neoplasms of the pancreas and other tumoral intraepithelial neoplasms of pancreatobiliary tract: recommendations of verona consensus meeting. Ann Surg. 2016;263:162–177. 25775066 10.1097/SLA.0000000000001173 PMC4568174

[15] Roldan J Harrison JM Qadan M. Evolving trends in pancreatic cystic tumors: a 3-decade single-center experience with 1290 resections. Ann Surg. 2023;277:491–497. 34353996 10.1097/SLA.0000000000005142

[16] Pergolini I Sahora K Ferrone CR. Long-term risk of pancreatic malignancy in patients with branch duct intraductal papillary mucinous neoplasm in a referral center. Gastroenterology. 2017;153:1284–1294.e1. 28739282 10.1053/j.gastro.2017.07.019

[17] Sauvanet A Couvelard A Belghiti J. Role of frozen section assessment for intraductal papillary and mucinous tumor of the pancreas. World J Gastrointest Surg. 2010;2:352–358. 21160843 10.4240/wjgs.v2.i10.352 PMC2999199

[18] Fong ZV Zwart MJW Gorris M. Intraoperative pancreatoscopy during robotic pancreatoduodenectomy and robotic distal pancreatectomy for intraductal papillary mucinous neoplasm with involvement of the main pancreatic duct. Ann Surg Open. 2023;4:e283. 37601466 10.1097/AS9.0000000000000283 PMC10431574

[19] Ciprani D Frampton A Amar H. The role of intraoperative pancreatoscopy in the surgical management of intraductal papillary mucinous neoplasms of the pancreas: a systematic scoping review. Surg Endosc. 2023;37:9043–9051. 37907657 10.1007/s00464-023-10518-8

[20] Kaneko T Nakao A Nomoto S. Intraoperative pancreatoscopy with the ultrathin pancreatoscope for mucin-producing tumors of the pancreas. Arch Surg. 1998;133:263–267. 9517737 10.1001/archsurg.133.3.263

[21] de Jong DM Stassen PMC Groot Koerkamp B; European Cholangioscopy study group. The role of pancreatoscopy in the diagnostic work-up of intra-ductal papillary mucinous neoplasms: a systematic review and meta analysis. Endoscopy. 2022;55:25–35. 35668651 10.1055/a-1869-0180 PMC9767751

[22] Yang HY Kang I Hwang HK. Intraoperative pancreatoscopy in pancreaticoduodenectomy for intraductal papillary mucinous neoplasms of the pancreas: Application to the laparoscopic approach. Asian J Surg. 2022;46:166–173. 35331591 10.1016/j.asjsur.2022.03.003

[23] Chavez JA Chen W Freitag CE. Pancreatic frozen section guides operative management with few deferrals and errors. Arch Pathol Lab Med. 2022;146:84–91. 33769446 10.5858/arpa.2020-0483-OA

[24] Kooby DA Lad NL Squires MH 3rd. Value of intraoperative neck margin analysis during Whipple for pancreatic adenocarcinoma: a multicenter analysis of 1399 patients. Ann Surg. 2014;260:494–501; discussion 501. 25115425 10.1097/SLA.0000000000000890

[25] Crippa S Ricci C Guarneri G. Improved survival after pancreatic re-resection of positive neck margin in pancreatic cancer patients. A systematic review and network meta-analysis. Eur J Surg Oncol. 2021;47:1258–1266. 33487492 10.1016/j.ejso.2021.01.001

[26] Nitschke P Volk A Welsch T. Impact of intraoperative re-resection to achieve r0 status on survival in patients with pancreatic cancer: a single-center experience with 483 patients. Ann Surg. 2017;265:1219–1225. 27280512 10.1097/SLA.0000000000001808

[27] Arnelo U Siiki A Swahn F. Single-operator pancreatoscopy is helpful in the evaluation of suspected intraductal papillary mucinous neoplasms (IPMN). Pancreatology. 2014;14:510–514. 25287157 10.1016/j.pan.2014.08.007

[28] Vehvilainen S Fagerstrom N Valente R. Single-operator peroral pancreatoscopy in the preoperative diagnostics of suspected main duct intraductal papillary mucinous neoplasms: efficacy and novel insights on complications. Surg Endosc. 2022;36:7431–7443. 35277769 10.1007/s00464-022-09156-3 PMC9485081

